# The Influence of Different Partial Pressure on the Fabrication of InGaO Ultraviolet Photodetectors

**DOI:** 10.3390/s16122145

**Published:** 2016-12-15

**Authors:** Sheng-Po Chang, Li-Yang Chang, Jyun-Yi Li

**Affiliations:** Institute of Microelectronics & Department of Electrical Engineering, Center for Micro/Nano Science and Technology, Advanced Optoelectronic Technology Center, National Cheng Kung University, Tainan 70101, Taiwan; leon50108@yahoo.com.tw (L.-Y.C.); z823040@gmail.com (J.-Y.L.)

**Keywords:** InGaO, photodetector, oxygen partial pressure

## Abstract

A metal–semiconductor–metal ultraviolet photodetector has been fabricated with a radiofrequency (RF)-sputtered InGaO thin film. Results for the devices fabricated under different oxygen partial pressure are here in discussed. Under low oxygen partial pressure, the devices work in the photoconductive mode because of the large number of subgap states. Therefore, the devices exhibit internal gain. These defects in the films result in slow switching times and lower photo/dark current ratios. A higher flow ratio of oxygen during the sputtering process can effectively restrain the oxygen vacancies in the film. The responsivity of the photodetector fabricated under an oxygen flow ratio of 20% can reach 0.31 A/W. The rise time and decay time can reach 21 s and 27 s, respectively.

## 1. Introduction

Ultraviolet (UV) photodetectors have drawn increasing attention owing to their multiple applications, such as chemical analysis, water purification, flame detection, and secure communications [1,2,3]. According to the cutoff wavelength, the devices can be divided into visible-blind photodetectors (λ ≤ 400 nm) and solar-blind photodetectors (λ ≤ 280 nm). For instance, solar-blind sensors applied in the military can be used in missile warning systems and therefore have no natural (sun) false alarm. Visible-blind sensors can be used for studying the ozone layer where UV radiation is intense.

Recently, wide bandgap semiconductors such as ZnO, GaN, and TiO_2_ have been commonly investigated owing to their promising advantages such as high sensitivity at room temperature [4,5,6,7,8,9]. However, indium–gallium–oxide (IGO) is not commonly reported in the literature. IGO is a metal–oxide material with potential. The bandgap of In_2_O_3_ is approximately 2.9 eV, approaching the visible-blind region. We can broaden the bandgap of In_2_O_3_ by doping with Ga_2_O_3_ [10], which is usually used in solar-blind photodetectors [11,12] and has a wide bandgap energy of approximately 4.9 eV. With Ga_2_O_3_, the oxygen deficiencies of In_2_O_3_ can also be restrained because of the strong bonding between gallium atoms and oxygen atoms.

Wenckstern et al. investigated the optical properties of In_x_Ga_1−x_O thin films and characteristics of Schottky contacts thereon [13]. Chang et al. reported amorphous IGO UV photodetectors prepared by co-sputtering [14]. The amount of oxygen vacancies in the film often play an important role in the performance of the devices and are always determined by the oxygen partial pressure during the process. However, to date there has still been no research on the influence of different partial pressures on the fabrication of IGO photodetectors. In this study, IGO UV photodetectors based on a metal–semiconductor–metal (MSM) structure have been fabricated under different oxygen partial pressures by radiofrequency (RF) magnetron sputtering at room temperature. The performance of the devices and the mechanism of their photoresponse characteristics are studied in detail.

## 2. Materials and Methods

**Figure 1:**
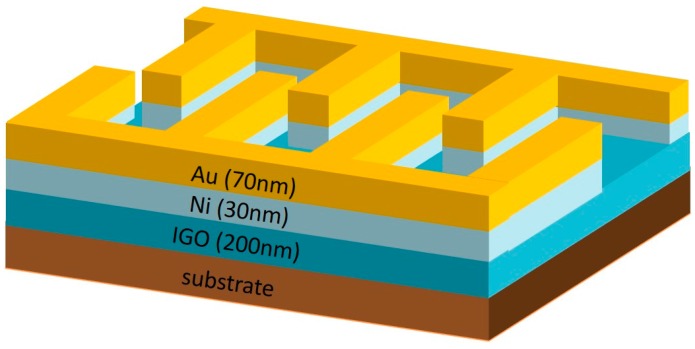
Schematic structure of a metal–semiconductor–metal (MSM) photodetector.

Quartz substrates were cleaned in an ultrasonic bath with acetone, methanol, and deionized water. IGO films of thickness 200 nm were deposited on the substrate by RF magnetron sputtering at room temperature, using an IGO ceramic sputtering target (atomic ratio In:Ga = 9:1, 99.99% pure). The RF power was 100 W. The chamber was evacuated below 10^−6^ torr. The pressure was 5 mtorr during the process, and the gas flow ratios of O_2_/Ar were 0%, 5%, 10%, and 20%. Interdigital Ni/Au electrodes were deposited by thermal evaporation onto the IGO thin films. With the Ni layer, the contact between the semiconductor and metal was improved. The fingers were 0.1 mm in width, 1.2 mm in length, and the inter-finger distance was 0.2 mm. A schematic of an IGO MSM photodetector is illustrated in Figure 1. X-ray diffraction (XRD) of the films was observed using a Rigaku X-ray diffractometer. The absorption coefficient spectra were measured by UV-visible scanning spectrophotometry. The surface smoothness was characterized by an atomic force microscope (AFM). The current–voltage characteristic was measured using an Agilent B1500 semiconductor parameter analyzer. The photoresponse of the photodetector was measured with a monochromator equipped with a 250 W Xe lamp.

## 3. Results and Discussion

**Figure 2:**
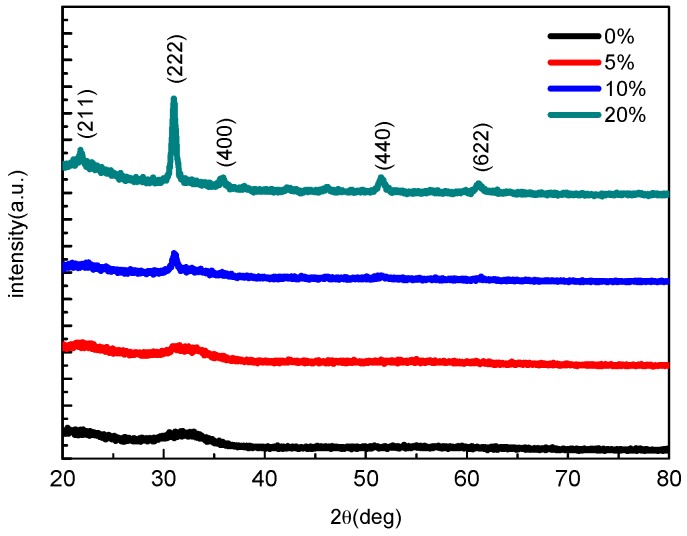
X-ray diffraction (XRD) patterns of the indium–gallium–oxide (IGO) films with different oxygen partial pressure by radiofrequency (RF) magnetron sputtering.

Figure 2 shows XRD patterns of the IGO films. The film is amorphous under 0% oxygen. Numerous peaks are observed from the samples under higher oxygen partial pressure. The peak intensities increased and the full width at half-maximum (FWHM) decreased as the oxygen partial pressure increased, indicating that the crystalline qualities are improved. The peaks (222), (440), (400), and (622) observed in IGO film fabricated under 20% oxygen partial pressure are consistent with the phase of In_2_O_3_. It is obvious that the 20% IGO film shows a polycrystalline phase.

**Figure 3:**
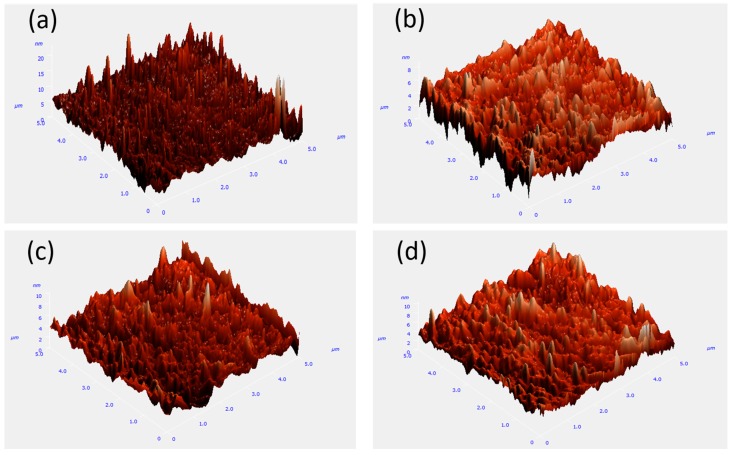
Atomic force microscope (AFM) images of the IGO films sputtered under (**a**) 0%; (**b**) 5%; (**c**) 10%; and (**d**) 20%.

**Figure 4:**
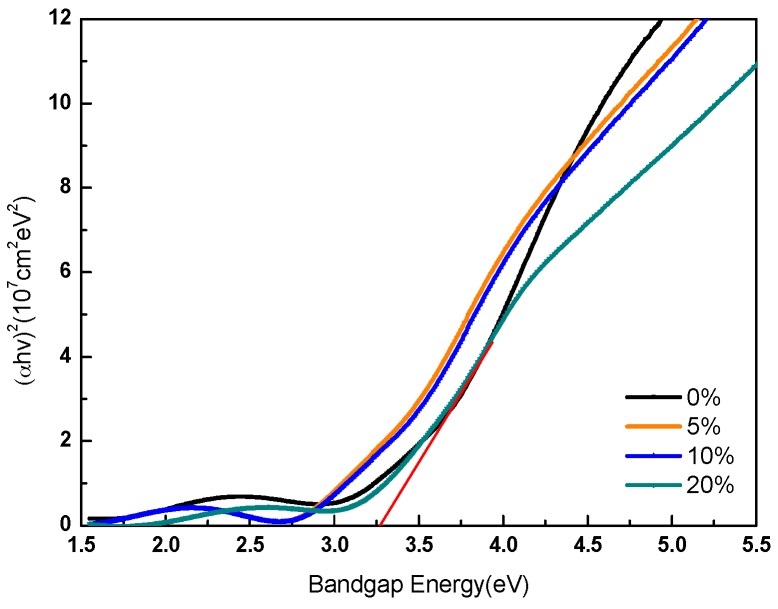
Absorption coefficient spectra of IGO films.

Figure 3 shows AFM images of the IGO films. The scanning areas were 5 μm × 5 μm. The root mean square surface roughness of the 5%, 10%, and 20% films were 0.879 nm, 1.097 nm, and 1.088 nm, respectively. The smooth surface of the films lead to lower surface density of states, which is beneficial for the performance of the devices [15]. Figure 4 shows optical absorption spectra of the IGO films. The bandgap of IGO is approximately 3.1–3.3 eV. The bandgap of the sample fabricated under 20% rises to 3.3 eV, with strong absorption for photons with wavelength shorter than 380 nm. It can be ascribed to the larger binding energy of GaO, and the formation of GaO decreases the conductivity of the film. The excess oxygen could bind with In atoms stably, leading to the apparent crystalline phase. The results of electrical measurement can also prove this deduction. Transmittance spectra of different partial pressure are shown in Figure S1 of the Supplementary Materials.

**Figure 5:**
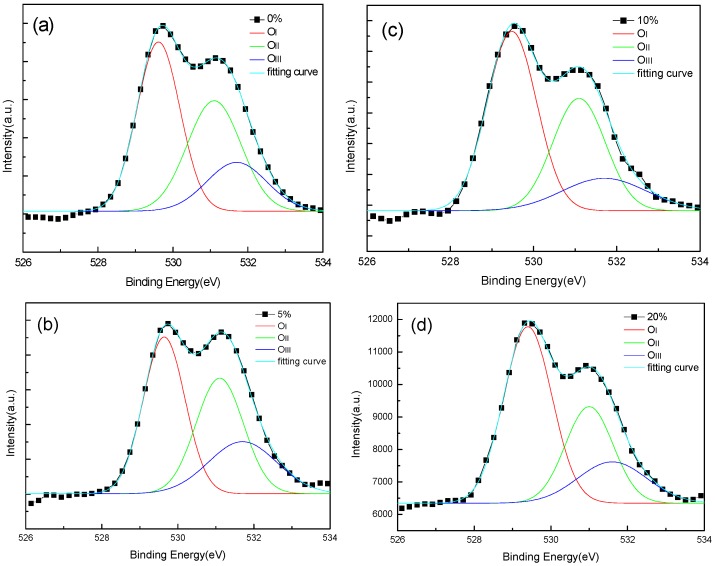
X-ray photoelectron spectroscopy (XPS) spectra of O_1s_ of IGO film with (**a**) 0%; (**b**) 5%; (**c**) 10%; and (**d**) 20%.

The chemical composition of the IGO films was investigated by X-ray photoelectron spectroscopy (XPS) measurements. In order to differentiate the XPS measurements, the typical O_1s_ peak was divided into three peaks through Gaussian fitting, as shown in Figure 5. The deconvoluted results of the O_1s_ peaks exhibited a peak at around 529.6 eV (O_I_), which is ascribed to the O_2_^−^ ions among metal oxides; a second peak, around 531.1 eV (O_II_), is usually attributed to the oxygen vacancies in the film; the third peak, located at about 531.7 eV, is related to weakly bound oxygen species on the film surface, such as CO_3_, OH^−^, or adsorbed O_2_ [16]. The relative ratio of O_II_/O_total_ are 36.4%, 35.2%, 33.8%, and 28.8% for pO_2_ = 0%, 5%, 10%, and 20%, respectively. As the partial oxygen pressure increases, the oxygen in the chamber tends to fill up the deficiencies in the IGO crystal lattice, leading to remarkable reduction in the vacancies in the film.

**Figure 6:**
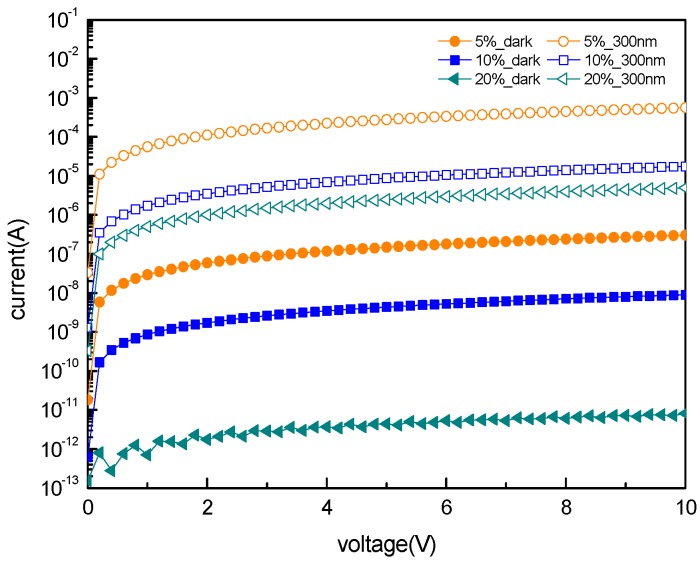
I-V characteristics for Au/IGO/Au, with varying oxygen partial pressure.

The current–voltage characteristics of the IGO photodetector are shown in Figure 6. The dark current of the devices fabricated under pO_2_ = 5% and pO_2_ = 10% changes linearly with bias from −10 V to 10 V, as shown in Figure S2a and Figure S2b of the Supplementary Materials. This indicates that they are ohmic devices. The dark currents for pO_2_ = 5%, 10%, and 20% are 3.03 × 10^−7^ A, 8.93 × 10^−9^ A, and 8.1 × 10^−12^ A at 10 V bias, respectively. The small dark current of the 20% fabricated device is attributed to the high Schottky barrier height and the reduction of the oxygen vacancies in the film. The Schottky characteristic is proved by Figure S3 in the Supplementary Materials. During the photocurrent measurement, the devices were illuminated under 300 nm UV light. The photocurrents for pO_2_ = 5%, 10%, and 20% are 5.63 × 10^−4^ A, 1.74 × 10^−5^ A, and 4.99 × 10^−6^ A, respectively.

**Figure 7:**
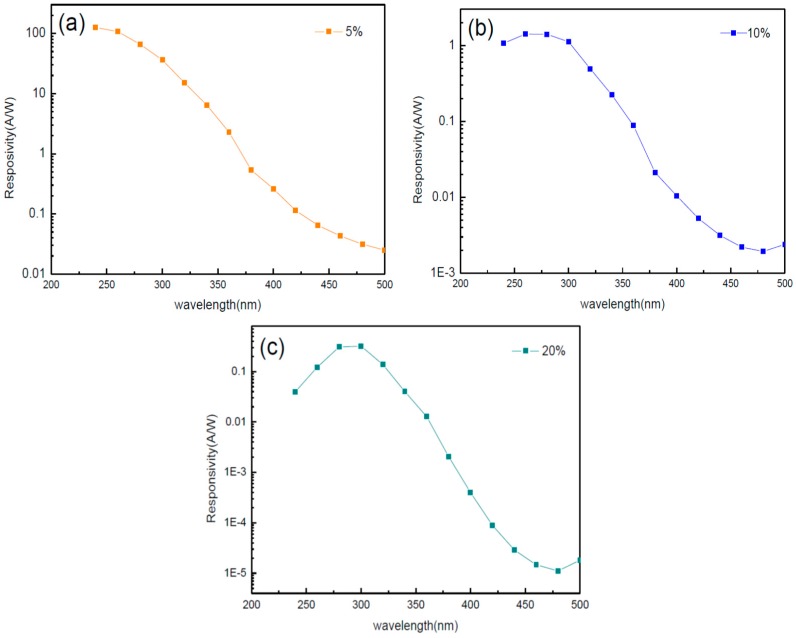
Responsivity of the devices with (**a**) 5%; (**b**) 10%; and (**c**) 20%.

Figure 7 shows the characteristic photoresponse spectra of the photodetector at 10 V bias. The responsivity can be expressed as:

(1) $$R=g\frac{\eta q}{h\upsilon },$$

where *η* is the quantum efficiency, *q* is the electron charge, *h* is Plank’s constant, and *υ* is the frequency of incident light.

Calculated from Equation (1), the value of *η* is over 100%, which confirms that there is internal photoconductive gain exhibited by the 5% and 10% fabricated devices. Photoconductive gain (*g*) [17] can also be defined as:

(2) $$g=\frac{N_{el}}{N_{ph}}=\frac{\tau_{decay}}{t_{tr}},$$

where *N_el_* is the number of electrons collected per unit time, *N_ph_* is the number of absorbed photons per unit time, *τ_decay_* is the hole (minority) lifetime, and *τ_tr_* is the electron transit time.

Therefore, we know the trapping mechanism plays an important role in the high photoconductive gain [18]. Under 20% oxygen partial pressure, the trapping phenomenon is modified and its maximum responsivity under 300 nm illumination is approximately 0.31 A/W. Table 1 reveals the responsivity of different sensing films fabricated by different methods. As shown in Table 1, the maximum responsivity obtained in this study represents an improvement compared with previous devices.

**Figure 8:**
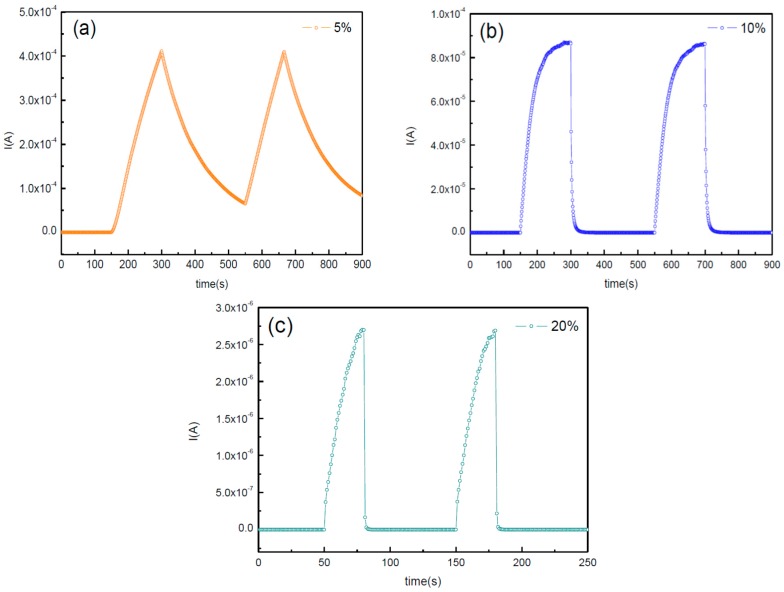
Time-dependent photoresponse of the IGO photodetector with (**a**) 5%; (**b**) 10%; and (**c**) 20%.

Figure 8 shows the photocurrent rise time and decay time under application of 10 V bias. The 5% and 10% device are compared in the same cycles. After the illumination with 300 nm UV light for 150 s, the photocurrent of 10% device can reach to maximum current and become stable. When the UV illumination is turned off, the electrons and holes of the 10% device can recombine in about 200 s. Nonetheless, under the 150 s illumination, the 5% device is still at the rising stage. Also, the recombination time of the device is extremely long. This is attributed to the existence of subgap states formed by oxygen vacancies in the film. These subgap states hinder the speed of electron excitation upon UV illumination and recombination when the light is turned off. On the contrary, it can be observed that the 20% fabricated photodetector shows fast response times, owing to the short lifetime of the carriers. Hence, the rise time and decay time are both apparently improved by increasing oxygen flow. The rise time is defined as the time in which the current rises from 10% to 90% of the maximum photocurrent. The decay time is the time in which the current decreases from the maximum photocurrent to the original value. The rise/decay times of pO_2_ = 20% are only 21/27 s.

**Figure 9:**
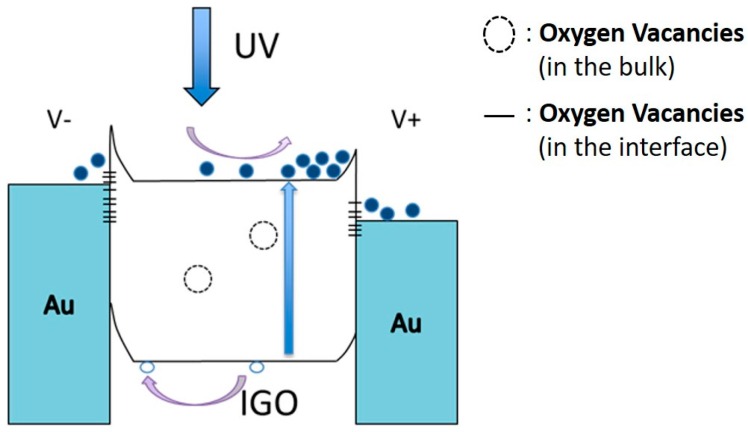
Band diagram of the MSM IGO photodetector under UV light.

Based on the above experimental results and analysis, the band diagram is shown in Figure 9 to explain the whole mechanism. For 5% and 10% fabricated devices, although there is sufficient barrier height between Au and the IGO films, the large number of positively charged oxygen vacancies in the interface narrows the depletion region, increasing the probability of electronic transport by tunneling [24]. As a consequence, the dark current is usually larger in the ohmic-type devices. During the illumination, the additional photon energy separates the electrons and holes. The excited electrons tend to move toward the conduction band; however, the vacancies in the film might capture and trap electrons. The large dark current and the trapping mechanism lead to a lower photo/dark current ratio. For the 20% fabricated device, the defects in the film are restrained, so it becomes a Schottky-type device. During the dark current measurement, the transport process is dominated by thermionic emission of carriers through the barrier; hence, the dark current is apparently reduced. Under UV illumination, the redistribution of space charge gives rise to an increase in positive charge density in the depletion region, causing the barrier to shrink [25]. Therefore, the tunneling probability increases. Both small dark current and the increasing tunneling probability lead to relatively high photocurrent/dark current ratio.

## 4. Conclusions

In summary, we investigated the photodetector performance of IGO film as a function of RF-sputtered oxygen partial pressure. For a lower oxygen partial pressure, the devices acted as ohmic-type photodetectors with high internal gain. When deposited at a higher oxygen partial pressure, the devices performed as Schottky-type photodetectors with lower dark current because of the reduction of deficiencies in the film. The responsivity reached 0.31 A/W. In addition, the photoresponse times of the devices were significantly improved by raising the oxygen partial pressure.

## Figures and Tables

**Table 1:** Maximum responsivity compared with different MSM ultraviolet photodetectors.

Sensing Film	Method	Responsivity (A/W)	Reference
MgZnO	Sputter	0.043	[19]
Mg_0.18_Zn_0.82_O	Sol-gel	0.27	[20]
IGO	Co-sputter	6.9 × 10^−5^	[14]
Ga_2_O_3_	Sol-gel	8 × 10^−5^	[21]
Ga_2_O_3_	MBE	0.037	[22]
Ga_2_O_3_	LMBE	0.05	[23]
In_0.9_Ga_0.1_O	Sputter	0.31	This study

## Supplementary Materials

The Supplementary Materials are available online at http://www.mdpi.com/1424-8220/16/12/2145/s1.
