# Supplementary material for: The Influence of Different Partial Pressure on the Fabrication of InGaO Ultraviolet Photodetectors
Source: Sensors (Basel). 2016 Dec 15;16(12):2145. doi: 10.3390/s16122145 (PMC5191125; doi:10.3390/s16122145)
Supplement: Supplementary file 1 [file sensors-16-02145-s001.pdf]

Supplementary Materials: The Influence of Different Partial Pressures on the Fabrication of InGaO Ultraviolet Photodetectors

Sheng-Po Chang, Li-Yang Chang and Jyun-Yi Li

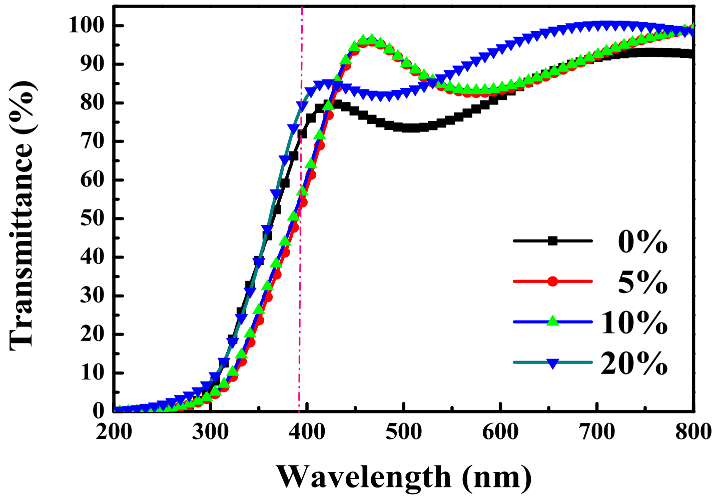

Figure S1. Transmittance spectra of different partial pressure on IGO films.

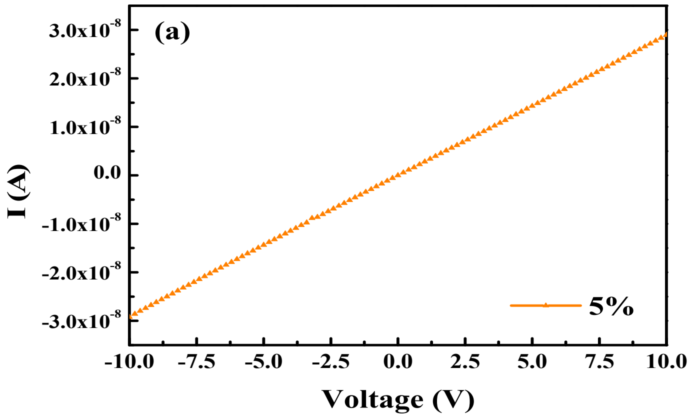

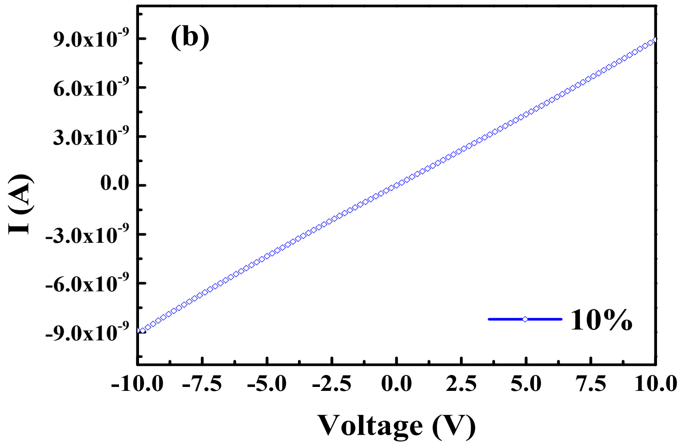

Figure S2. The linear dark I–V characteristics curve of the IGO thin films MSM photodetector fabricated under pO_2 = (**a**) 5% (**b**) 10% in linear scale.

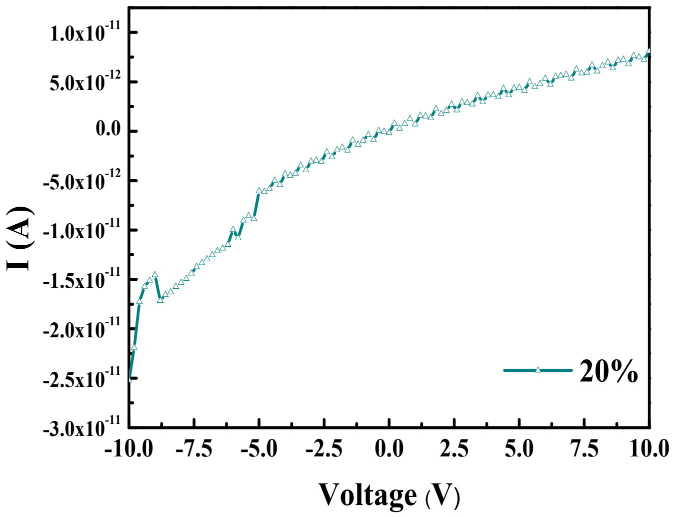

Figure S3. The Schottky dark I–V characteristics curve of the IGO thin films MSM photodetector fabricated under pO_2 = 20% in linear scale.
